# Hydrothermal Synthesis of Ag Thin Films and Their SERS Application

**DOI:** 10.3390/nano12010136

**Published:** 2021-12-31

**Authors:** Nikolay P. Simonenko, Andrey G. Musaev, Tatiana L. Simonenko, Philipp Yu. Gorobtsov, Ivan A. Volkov, Alexander A. Gulin, Elizaveta P. Simonenko, Vladimir G. Sevastyanov, Nikolay T. Kuznetsov

**Affiliations:** 1 Kurnakov Institute of General and Inorganic Chemistry of the Russian Academy of Sciences, 31 Leninsky pr., 119991 Moscow, Russia; egorova.offver@gmail.com (T.L.S.); phigoros@gmail.com (P.Y.G.); ep_simonenko@mail.ru (E.P.S.); vg_sevastyanov@mail.ru (V.G.S.); ntkuz@igic.ras.ru (N.T.K.); 2 Moscow Institute of Physics and Technology, National Research University, 9 Institutskiy per., 141701 Dolgoprudny, Russia; musaev.ag@mipt.ru (A.G.M.); volkov256@yandex.ru (I.A.V.); 3 N.N. Semenov Federal Research Center for Chemical Physics of the Russian Academy of Sciences, 4 Kosygina Street, Building 1, 119991 Moscow, Russia; aleksandr.gulin@phystech.edu

**Keywords:** Ag film, silver nanoparticles, hydrothermal synthesis, surface-enhanced Raman spectroscopy, methylene blue

## Abstract

In this article, a facile, one-step method for the formation of silver thin-film nanostructures on the surface of Al_2_O_3_ substrates using the hydrothermal method is proposed. The dependence of the SERS effect intensity of the formed films during the detection of methylene blue (MB) low concentrations on the synthesis conditions, additional temperature treatment, and laser radiation wavelength (532 and 780 nm) in comparison with similar dye films on commercial SERS substrates is shown. The detection limit of the analyte used for the indicated lasers is estimated. The effect of the synthesis temperature on the particle size, crystal structure, and microstructure features of the obtained thin films based on silver nanoparticles is demonstrated. Using spreading resistance microscopy, the interface between the substrate and Ag particles is studied, and the dependence of the size of the corresponding gap between them and the nature of microstructural defects on the parameters of hydrothermal treatment of reaction systems in the presence of Al_2_O_3_ substrates is shown. As a result of the study, the factors associated with the properties of the obtained SERS substrates and the parameters of recording the spectra, which affect the amplification factor of the spectral lines intensity of the analyte, are revealed.

## 1. Introduction

As is known, surface-enhanced Raman spectroscopy (SERS) has proven to be an effective high-sensitivity method of chemical analysis since its discovery, as far as this approach makes it possible to amplify Raman signals by several orders of magnitude when implementing electromagnetic and chemical effects [1]. In this case, charge transfer between the adsorbed analyte molecules and the surface of metal particles occurs, as well as an amplification of the electromagnetic field with excitation of the localised plasmon resonance on the metal surface. Signal amplification occurs when analyte molecules are located in the spaces between neighbouring metal nanoparticles (so-called ‘hot spots’) [2]. SERS is used as a useful tool in various applications—analytical chemistry, atmospheric monitoring, safety control, biodetection, biomedicine, etc. [2,3].

As a rule, surface-enhanced Raman spectroscopy uses substrates whose surface is modified with silver, gold, or copper nanostructures with different microstructural characteristics [4,5,6,7,8,9,10,11,12]. Recently, much of researchers’ attention has also been directed toward studying the processes of obtaining composite SERS-active films—Ag@nanoAu [4], Ag nanocubes@poly(dimethylsiloxane) [13], Ag/TiO_2_ [14], MoO_3-x_/Ag [15], b-cyclodextrin-modified Ag [16], Au/Ag and Au/Ni [7,17], Ag particles/Ag-Zr alloy [18], Mo-Ag [19], Ag/AgBr/ZnO [20], ZnO/Ag 3D nanocomposites [21,22], ZnO/Au [23], MnO_2_/Au [24], Au/ZrO_2_ [25], rGO-Ag [26], or Ag/C [27]. In some cases, metal-free SERS substrates, particularly based on MoS_2_, are proposed [28]. Flexible substrates and membranes made of polymeric materials such as poly(dimethylsiloxane), polyimide, polyacrylonitrile, polyvinyl alcohol, etc. are also often used [13,17,18,19,29,30]. These nanostructures are obtained using a wide range of different methods—in situ formation at interfaces [31], thermal evaporation [32], solvent evaporation [33], one step galvanic displacement method which involves dipping [34], the Langmuir–Blodgett (LB) technique [35], convective assembly [36], chemical deposition [2,4,37], layer-by-layer assembly [38], vapor-phase deposition [5,39], spin casting [40], photochemical deposition [14,20]. Due to the wide variability and possibility to optimise different parameters, liquid-phase methods are the most convenient and simple for synthesising planar nanomaterials of different compositions of both metal and oxide [2,41,42,43]. Anisotropic and hierarchically organized SERS nanostructures are of particular interest today, since they often exhibit increased efficiency and selectivity for certain analytes [2,6,7,13,30,39,44], and one of the most convenient approaches to the synthesis of such planar nanomaterials with an ordered microstructure is the hydrothermal method [45,46,47,48,49,50,51]. The main advantages of this method lie in the possibility to finely control the morphology of the formed materials, as well as in ensuring reproducibility, uniformity, and high adhesion of the grown coatings on substrates of different types. One of the dynamically developing and promising approaches to the formation of coatings with an ordered structure, including those based on metal nanoparticles, are printing technologies [52] that improve the targeted application of the material and the reproducibility of the process, which is very important in the fabrication of SERS-active nanostructures.

In this work, we proposed a simple, one-step method for obtaining thin-film Ag nanostructures on the surface of Al_2_O_3_ substrates using a hydrothermal method and found that they exhibit a more intense SERS signal, compared with commercial counterparts, when using methylene blue as an analyte and lasers with emission wavelengths of 532 and 780 nm.

## 2. Materials and Methods

### 2.1. Materials

Silver nitrate (AgNO_3_, 99.8%, Lenreactiv, Saint Petersburg, Russia), triethanolamine (C_6_H_15_NO_3_, 99%, Chimmed, Moscow, Russia), acetic acid (C_2_H_4_O_2_, 70% aqueous solution, Lenreactiv, Saint Petersburg, Russia), 2-propanol (C_3_H_8_O, 99.8%, Ekos-1, Moscow, Russia), and 2-butanol (C_4_H_10_O, 99%, Ekos-1, Moscow, Russia) were used as reagents in this study. Ag films were applied to the surface of Al_2_O_3_ substrates (VK-100, 99.6% Al_2_O_3_ content, C-Component, Moscow, Russia). When studying the intensity of SERS signals from the surface of hydrothermally grown Ag films, commercial analogs (Silver substrate ‘Randa S’, SERS-AG-35-1, Ato ID, Vilnius, Lithuania) were used for comparison. Methylene blue dye was used as an analyte in the SERS measurements.

### 2.2. Hydrothermal Synthesis of Ag Thin Films

**Figure 1:**
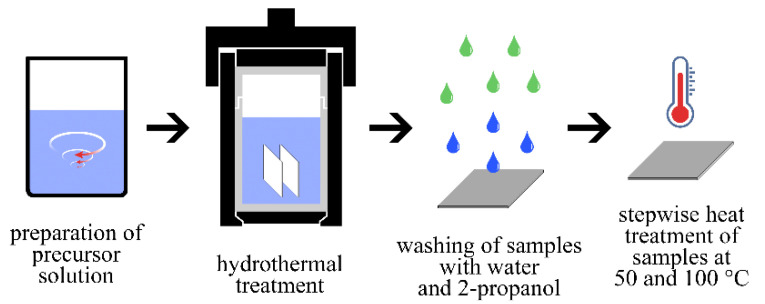
Scheme of the experiment.

Ag films were grown according to the scheme shown in Figure 1. The precursor solution in a typical experiment was prepared by dissolving 0.250 g of silver nitrate in 20 mL of distilled water, after which 1.280 g of triethanolamine was added under stirring, resulting in precipitation. Next, 0.4 mL of acetic acid solution was added to the reaction system until the precipitate dissolved completely, followed by 20 mL of 2-propanol addition. The concentration of silver cations (about 0.035 mol/L) and the content of other components in the reaction system were chosen to prevent an excessive rate of silver particle formation and immoderate amounts of solid phase formed. From the obtained solution of silver heteroligand complexes, an aliquot (10 mL) was taken and placed in a steel autoclave with a Teflon liner (a total vessel volume of 25 mL) where two pre-cleaned and degreased Al_2_O_3_ substrates were mounted vertically. The autoclave was then hermetically sealed and subjected to heating in a muffle furnace to 100, 120, and 140 °C at a rate of 5°/min, after which it was kept at a given temperature for 1 h and then naturally cooled to 25 °C along with the furnace. The heat treatment initiated the reduction of silver cations and the growth of metal nanoparticles on the surface of ceramic substrates. After cooling the reaction systems and extracting the substrates with the applied Ag films, the formed materials were washed with distilled water and 2-propanol, followed by drying at 50 °C for 2 h. For SERS measurements, the samples were additionally heat-treated at 100 °C to minimise the amount of residual organic components on their surface.

### 2.3. Application of Analyte Solutions to Substrates

The obtained films consisting of silver nanoparticles were investigated for the possibility of surface-enhanced Raman scattering using methylene blue as a test analyte. For this purpose, solutions with concentrations of this compound 10^−4^, 10^−5^, and 10^−6^ mol/L were prepared. A 4:1 mixture of 2-butanol and distilled water was used as a solvent. To detect the SERS effect and find the factors affecting the spectral line intensity gain, 0.1 µL solutions of the analyte with the concentration of 10^−4^ mol/L were applied to the samples of investigated coatings and ceramic substrate of aluminium oxide using a pipette dispenser. To evaluate the detection limit achieved through the SERS effect, solutions with a concentration of methylene blue 10^−4^, 10^−5^, and 10^−6^ mol/L were applied to the coating samples in a similar manner. The solvent contained in the solutions was then completely evaporated for 10 min, resulting in the formation of a solid phase of the analyte distributed in the structure of the metal coating.

### 2.4. Instrumentation

The thermal behaviour of the precursor solution was analysed using a combined DSC/DTA/TG analyser SDT-Q600 (TA Instruments, New Castle, DE, USA) in Al_2_O_3_ micro crucible (airflow 250 mL/min; sample weight 44 mg; 1st step—heating to 100 °C, 10°/min, holding at 100 °C for 15 min; 2nd step—heating to 1000 °C, 10°/min).

X-ray diffraction analysis of the obtained films and the used Al_2_O_3_ substrates was performed on a D8-Advance diffractometer in the range of 2θ 20–80° (Bruker, Bremen, Germany, CuKα = 1.5418 Å, Ni-filter, E = 40 keV, I = 40 mA, integration time = 0.3 s/point, step = 0.02°). Analysis of the XRD spectra was carried out with the use of the Rietveld refinement method, implemented in X’Pert HighScore Plus software (PANalytical B.V., Almelo, The Netherlands).

The microstructure of as-grown Ag films was studied by scanning electron microscopy (Carl Zeiss NVision 40, Oberkochen, Germany) using secondary and backscattered electron detectors (accelerating voltage was 1 kV).

The surface of the obtained films was also studied by atomic force microscopy (AFM). As a result, data on the microstructure of the film surface as well as local electrophysical properties (electron work function and current–voltage curves) were obtained. These studies were performed on a Solver Pro-M scanning probe microscope (NT-MDT LLC, Zelenograd, Russia) in ambient conditions in semi-contact AFM, Kelvin probe force microscopy (KPSM) using ETALON HA-HR probes with a conductive coating based on W_2_C (resonance frequency ~366 kHz, spherical radius <35 nm) and in contact AFM and scanning spreading resistance microscopy modes using ETALON HA-C probes with a W_2_C-based conductive coating (force constant ~0. 26 N/m, spherical radius <35 nm). Grounding of the samples for measurements in the KPFM mode and applying a voltage to it for spreading resistance imaging was carried out as described in another study [53].

Raman spectroscopy was performed using a DXR Raman microscope (Thermo Fisher, Waltham, MA, USA). To work with the samples, a 10× objective was used, since it allowed the greatest reduction in the energy density of laser radiation falling on the investigated area. This made it possible to reduce the destruction of chemical bonds of the dye, which helped to slow down the reduction in the signal during measurements. Laser power, aperture width, exposure duration, and the number of shots were selected in such a way as to obtain the best signal amplification and signal-to-noise ratio. For a laser with a radiation wavelength of 532 nm, the power was 1 mW, the aperture was 25 mkm pinhole, the number of shots was 20, and the exposure was 1 s. For the 780 nm laser, the power was 20 mW, the aperture was 25 mkm pinhole, the number of shots was 30, and the exposure was 2 s. During the recording of the spectra, the result for all images was automatically averaged, and processing was immediately carried out to reduce the influence of fluorescence on the measurement result.

## 3. Results and Discussion

### 3.1. Characterisation of the Precursor Solution

**Figure 2:**
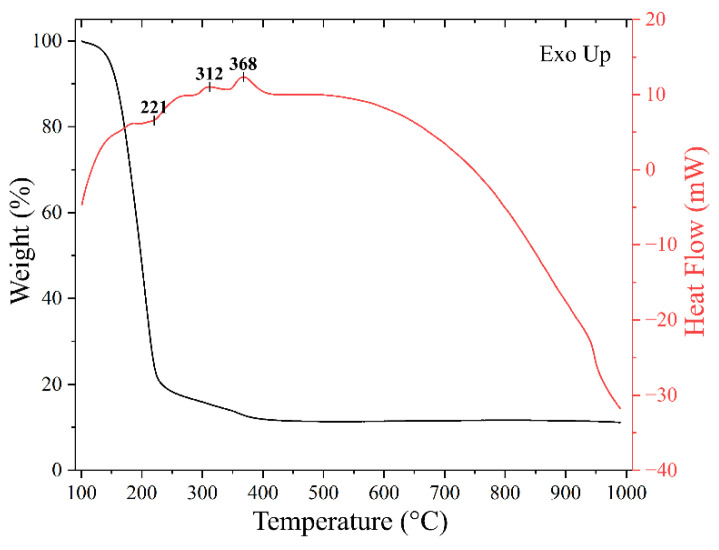
TGA and DSC curves for the precursor solution when heated in an air current in the temperature range 100–1000 °C.

The precursor solution used for the hydrothermal synthesis of Ag films was studied using synchronous thermal analysis under stage heating. In the first step (heating to 100 °C and holding at this temperature for 15 min), weight loss due to solvent evaporation was 83%. At further heating (Figure 2) there was a two-step weight loss—in the temperature intervals 100–230 °C (80.3%) and 230–400 °C (7.8%). During further heating, the change in the sample weight was insignificant, indicating complete removal of liquid components and decomposition of precursors up to 400 °C. In this case, the total weight loss of ink during heating in the temperature range of 25–400 °C was 99.49%. Thus, heat treatment of precursor solution film, applied to any substrate, can allow obtaining metal coatings, but at atmospheric pressure, in order to avoid admixture of organic components, heat treatment should be carried out in oxidising atmosphere at temperatures of about 350–400 °C, which can lead to significant coarsening of silver particles. The use of this precursor solution for the growth of Ag films under hydrothermal conditions can lead to a substantial reduction in the synthesis temperature while minimising the number of organic impurities in metal coatings and increasing the dispersity of the silver particles composing them.

### 3.2. Crystal Structure of As-Grown Ag Films

**Figure 3:**
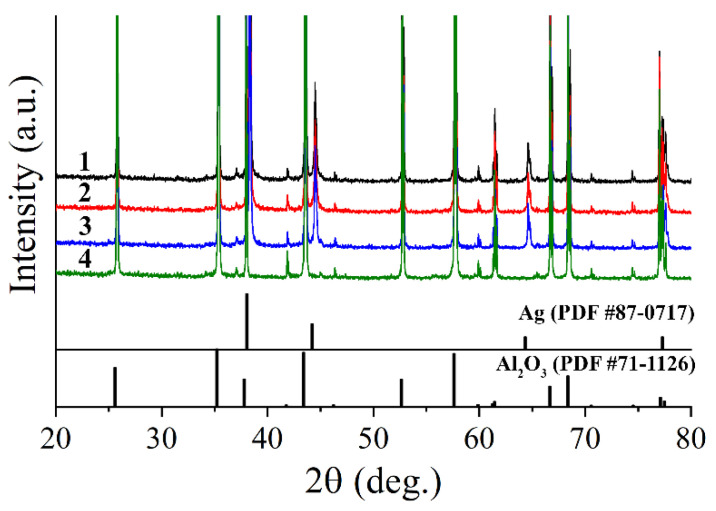
X-ray diffraction patterns of Ag films grown on the surface of Al_2_O_3_ substrates at different temperatures (1—100, 2—120, 3—140 °C) as well as a diffractogram of the original Al_2_O_3_ substrate (4).

As can be seen from X-ray diffraction patterns (Figure 3) of metal films grown in hydrothermal conditions on the surface of Al_2_O_3_ substrates under various conditions, in all cases, silver coatings were formed, having a characteristic set of reflexes corresponding to the cubic crystal lattice (space group Fm-3m), which agrees well with the literature data (PDF #87-0717). Sufficiently high intensity of reflexes of the substrate material (Al_2_O_3_, rhombohedral crystal lattice, space group R-3c, PDF #71-1126) indicates a thin-film structure of the formed Ag films. Full-profile analysis of patterns made it possible to estimate the average size of the coherent scattering region (CSR) of silver particles composing the films, which was 70 ± 7, 86 ± 8, and 81 ± 8 nm at synthesis temperatures of 100, 120, and 140 °C, respectively. As can be seen, the dependence of the average CSR size on the synthesis temperature had an extreme nature, and the most highly dispersed film, according to XRD, was formed at a temperature of hydrothermal treatment of 100 °C. The calculated values of the crystal lattice parameters also had extreme dependence on the synthesis temperature (100 °C: a = b = c = 4.088(1) Å, 120 °C: a = 4.089(1) Å, 140 °C: a = 4.088(1) Å). The results also indicate that the grown Ag films did not contain any crystalline impurities (reagents, precursors, or by-products).

### 3.3. Microstructure of the Studied Ag Films

**Figure 4:**
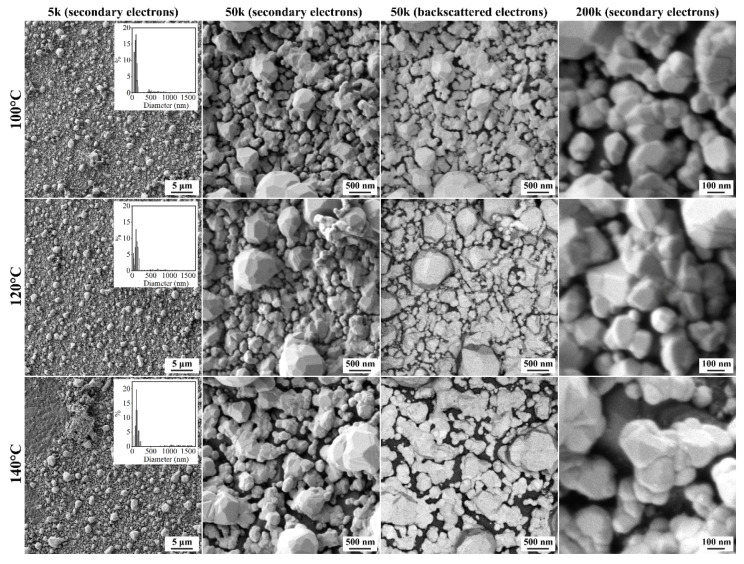
SEM micrographs of Ag films grown at different temperatures (using different detectors).

The microstructure of the Ag films grown under hydrothermal conditions was studied by scanning electron microscopy. As can be seen from the micrographs of the obtained metal films (Figure 4), they consist of particles with a bimodal size distribution. The average size of both small (100 → 110 → 130 nm) and large particles (620 → 700 → 1350 nm) increased with increasing synthesis temperature (100 → 120 → 140 °C). According to the backscattered electron detector data, gaps between silver particles were well observed, the average size of which increased significantly (30 → 40 → 80 nm) as the hydrothermal treatment temperature increased. As can be seen, when increasing the synthesis temperature from 120 to 140 °C, there was a sharp (twofold) jump in the average value of the gap between the silver particles. In addition, for the Ag film obtained at 140 °C, a significant number of defects in the form of its delamination from the surface of the Al_2_O_3_ substrate was observed—probably, the adhesion deteriorated due to a sharp increase in the gaps between the metal particles. Thus, it can be assumed that at the first stage, films of nanoscale particles were grown on the surface of Al_2_O_3_ substrates, which agglomerated and enlarged at increasing temperature with the formation of microscale structures, which led to the appearance of appropriate gaps and defects in the form of delamination.

**Figure 5:**
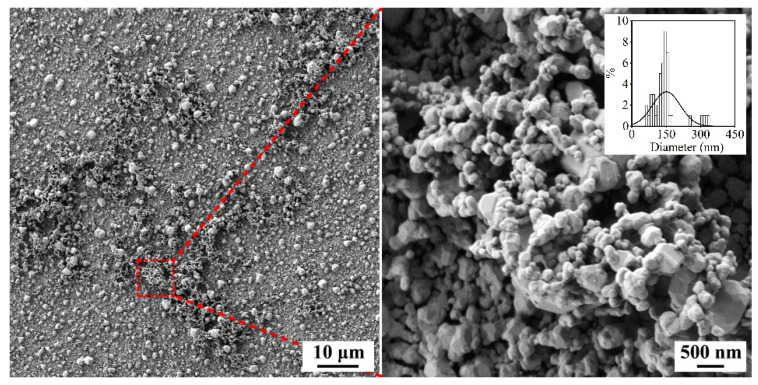
SEM micrographs of the dark area of the Ag-film surface grown in hydrothermal conditions at 100 °C.

When studying the obtained Ag films using optical microscopy (in particular, during SERS measurements), it was discerned that there were a few dark regions with sizes of about several tens of micrometres on their surface. A more detailed study of these areas using scanning electron microscopy showed (Figure 5) that these areas were clusters of relatively small particles with an average size of about 150 nm, organised into highly porous agglomerates.

**Figure 6:**
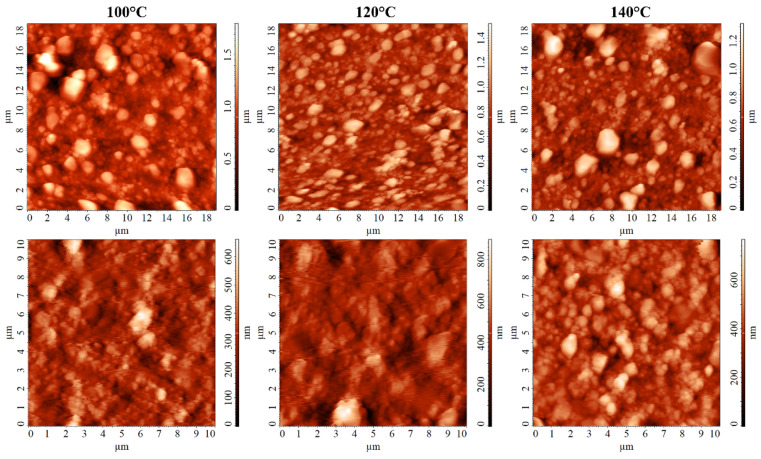
AFM micrographs of Ag films grown at different temperatures (**top**—the main surface; **bottom**—dark areas).

The microstructure of the grown Ag films was also studied using AFM in the contact and semi-contact modes. From the micrographs showing the surface topography of the materials (Figure 6), it is clear that the AFM results agree well with the SEM data. Bimodal particle size distribution was observed for all samples: both arrays of particles between 250 and 400 nm in size and particles from 1 micrometre and larger (but no larger than 3 μm) were observed. It can be seen that as the synthesis temperature increased, there was a tendency for the number of larger particles to increase. A study of the microstructure of the dark areas showed that even though there were still large particles in these areas, in the case of hydrothermal treatment temperatures of 100 and 120 °C, they were almost entirely formed by smaller particles sized 250–400 nm. However, in the case of the film grown at 140 °C, a noticeable number of microsized particles were found in the dark regions, which is further evidence of the trend toward an increasing proportion of large formations with increasing synthesis temperature. Additionally, in the topographic images for the Ag film formed at 140 °C, areas corresponding in their microstructure to the Al_2_O_3_ substrate were observed.

**Figure 7:**
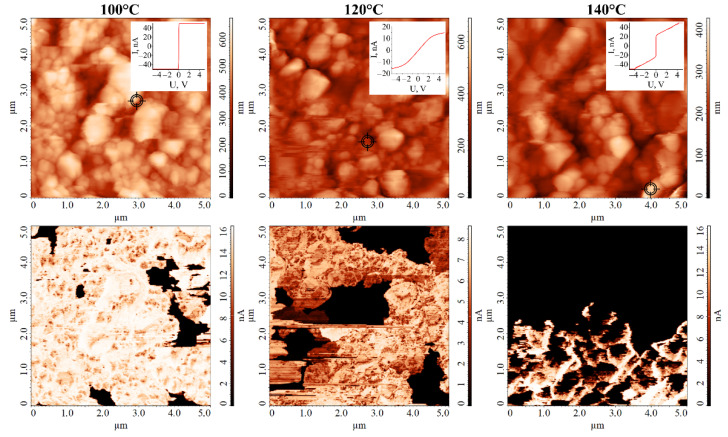
AFM microphotographs of Ag films grown at different temperatures (**top**—surface relief of the films; **bottom**—the corresponding current distribution maps; **inset**—current–voltage curves obtained at the specified points).

In order to study Ag particles distribution on the surface of ceramic substrates in more detail, we used scanning spreading resistance microscopy, through which a potential difference was created between the studied sample and the probe, and the intensity of the resulting current was measured. Due to the large differences in the electrophysical properties of silver and aluminium oxide, this technique makes it possible to obtain high-contrast maps of the mutual distribution of these materials. From the topographic images and maps of current distribution over the surface of the materials studied (Figure 7), it is clearly seen that an increase in the synthesis temperature led to an increase in the area of the Al_2_O_3_ substrate, where there were no silver particles (dark areas on the maps of current distribution). If, in the case of Ag film formed at 100 °C, individual small areas up to 1 μm long (and much less than 1 μm^2^ in area) could be found, for the film grown at 120 °C, such areas were about 1–2 μm^2^ in area. In the case of the film obtained in hydrothermal conditions at 140 °C, the area of regions uncovered by silver particles became higher than 10 μm^2^. This confirms the tendency revealed when studying the microstructure of Ag films using scanning electron microscopy. In addition, typical current–voltage curves were recorded in the spreading resistance microscopy mode at the indicated points on the surface of silver coatings. It should be noted that at current strengths of about 15–20 nA, even ohmic contacts often begin to show nonlinearity, which is associated with the design features of the atomic force microscope. The maximum detectable current value, in this case, is 50 nA. In our case, for all investigated silver films at current strengths below 10 nA, direct dependence of current strength on voltage typical of ohmic contact was observed, and for materials obtained at temperatures of 100 °C and 140 °C, this dependence continued to behave this way at higher currents (for Ag film obtained at 140 °C, only the slope angle changed). Moreover, for this film, even in the linear section, the slope angle of the straight line was much lower than for the other samples. This fact, as well as the significantly lower value of the achieved current strength, indicates that this coating has significantly lower electrical conductivity, although it is metallic.

**Figure 8:**
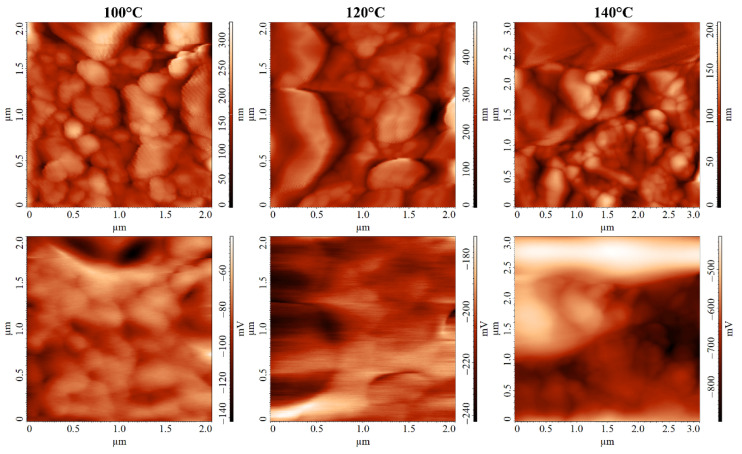
AFM micrographs of the surface of Ag films grown at different temperatures (**top**—film surface topography; **bottom**—the corresponding maps of surface potential distribution).

In addition to spreading resistance microscopy, Kelvin probe force microscopy was used to study the local electrophysical properties of the grown Ag films. From the obtained maps of the surface potential distribution (Figure 8), it can be seen that despite quite large height differences (up to 500 nm) between the silver particles, the distribution of the surface charge was quite uniform. An area on the scan for the sample obtained at 140 °C was noticeable, where the surface potential was almost 200 mV higher than for other areas on the scanned surface. Additionally, the topographic image shows that this area differed greatly in morphology from the rest of the scanned area, suggesting that it refers to the uncovered surface of the Al_2_O_3_ substrate, where the static charge accumulates. Using images obtained by scanning in KPFM mode, the values of electron work function were calculated to be 4.831, 4.941, and 5.015 eV for Ag films grown at 100, 120, and 140 °C, respectively. In all cases, the values of the electron work are higher than the reference values for silver, which can be explained by the high dispersity of metal particles. These values indicate that the intrinsic conductivity of the material may decrease with increasing synthesis temperature due to an increase in the Fermi energy.

### 3.4. Study of the SERS Effect When Detecting Methylene Blue

**Figure 9:**
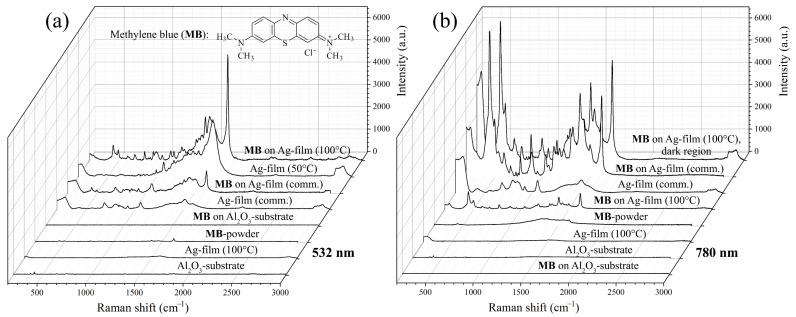
Raman and SERS spectra of the materials studied, obtained using lasers with an emission wavelength of 532 (**a**) and 780 nm (**b**); the indicated temperature values refer to the conditions of additional heat treatment of the Ag film in air.

The films formed under hydrothermal conditions were further studied for surface-enhanced Raman scattering when detecting methylene blue. The results of preliminary measurements showed that the silver films grown at 120 °C contained in their structure a sufficiently large number of organic components, which agrees with the results of the spreading resistance microscopy and significantly complicates their application in the detection of the analyte at low concentrations. Metal films obtained at 140 °C were characterised by a fairly low amplification factor of the intensity of methylene blue spectral lines, which is probably due to the peculiarities of the microstructure of this material (larger particles and the presence of defects in the form of silver film delamination). At the same time, the Ag film grown at the minimum temperature (100 °C) in the range under consideration exhibited the most intense SERS effect. As can be seen from the Raman spectra (Figure 9a) recorded using a laser with an emission wavelength of 532 nm, additional heat treatment of this film at 100 °C in air led to a significant reduction in the number of residual organic components contained on its surface after synthesis, washing, and drying at 50 °C. A significant enhancement of spectral lines was observed for the methylene blue applied to the Ag-film surface at a concentration of 10^−4^ mol/L in comparison with both this substance in powder form and similar dye films on the surface of pure Al_2_O_3_ substrate and commercial SERS substrate. When the SERS effect was studied using a 780 nm laser (Figure 9b), there was also a significant increase in the intensity of spectral lines, compared with the signal from the powder and dye film on the surface of pure Al_2_O_3_ substrate. At the same time, the signal from the dark areas on the surface of the Ag film grown at 100 °C was also more intense, compared with the commercial substrate. Thus, the presence of porous clusters of small silver particles on the substrate surface led to an additional significant enhancement of the SERS effect when detecting methylene blue also using a laser with an emission wavelength of 780 nm.

**Figure 10:**
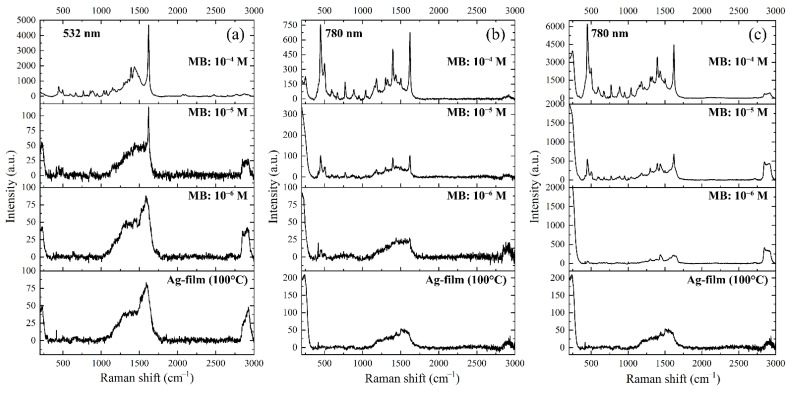
Raman spectra of Ag film (synthesis temperature—100 °C, additional heat treatment—100 °C) and SERS spectra of methylene blue deposited on its surface at different concentrations recorded from the main surface (**a**,**b**) and from the dark region of Ag film (**c**) using lasers with wavelengths of 532 and 780 nm.

Further, the Ag film formed under hydrothermal conditions at 100 °C and subjected to additional heat treatment at 100 °C in the air was used to estimate the detection limit of methylene blue achieved through the SERS effect. For this purpose, SERS spectra of this analyte at concentrations of 10^−4^, 10^−5,^ and 10^−6^ mol/L from the main surface (Figure 10a,b) and from the dark areas of the indicated silver film (Figure 10c) were obtained using lasers with different wavelengths of radiation. As can be seen from the results obtained, using a 532 nm laser, the minimum detection concentration of methylene blue was 10^−5^ mol/L. In contrast, when a laser with a wavelength of 780 nm was used, the detection limit of the indicated analyte for the main surface of the Ag film was also 10^−5^ mol/L, while for the dark areas, characterised by the presence of highly porous agglomerates of highly disperse silver particles, the minimum dye detection concentration was at 10^−6^ mol/L.

Thus, the observed more intense SERS effect upon the detection of methylene blue at low concentrations for an Ag film grown under hydrothermal conditions at 100 °C was due to a more uniform microstructure, smaller particle and CSR sizes, and a lower content of residual organic components.

We estimated the SERS enhancement factor (EF) for the methylene blue adsorbed on particles forming dark areas of Ag films under study based on the following most widely used definition for the average SERS EF [54].

EF = (I_SERS_/N_surf_)/(I_RS_/N_vol_), (1)

where I_SERS_ and I_RS_ are the intensities of a particular line of an analyte in the SERS and normal Raman (non-SERS) spectra measured under the same conditions, N_surf_ is the average number of adsorbed molecules in the scattering volume for the SERS experiment, and N_vol_ is the average number of molecules in the scattering volume for the non-SERS experiment. In calculations, we considered spectra of methylene blue measured by using a laser with an emission wavelength of 780 nm. To estimate the number of methylene blue molecules probed in the SERS experiment (i.e., N_surf_), the laser spot size, surface density of particles producing the enhancement, and surface density of molecules adsorbed on the metal must be known. In the performed experiments, the laser spot size was about 3.0 µm. Assuming the surface density of fine particles on the substrate to be about 2 × 10^9^ cm^–2^ (as estimated from SEM images of dark areas), the surface density of methylene blue molecules in the monolayer to be 10^14^ cm^–2^ [55], and around 7% surface coverage of the metal [54], approximately 6.7 × 10^5^ molecules (N_surf_) were probed in the SERS experiment. For the non-SERS experiment, the number of molecules (N_vol_) within the probed volume of a methylene blue solution (10^–3^ mol/L) dispensed on an alumina substrate was estimated at 4.2 × 10^8^. Substitution of N_surf_, N_vol_, and the intensities of methylene blue line located at about 1625 cm^–1^ (taken from the typical SERS spectrum acquired in the dark area of Ag film and the Raman spectrum of methylene blue solution) to Equation (1) yielded an EF value equal to about 1.8·10^5^.

## 4. Conclusions

A facile, one-step method of forming Ag thin-film nanostructures on the surface of Al_2_O_3_ substrates using a hydrothermal method was proposed. It was demonstrated that the silver films formed at 100 °C exhibited a more intense SERS effect when detecting low concentrations of methylene blue using 532 and 780 nm lasers, compared with both this substance in powder form and similar dye films on the surface of pure Al_2_O_3_ substrate and commercial SERS substrates. Using the 532 nm laser, the minimum detection concentration of methylene blue was 10^−5^ mol/L. In the case of the 780 nm laser, the detection limit of the analyte for the main surface of the Ag film was also 10^−5^ mol/L, and for the dark areas, whose microstructure was characterised by the presence of highly porous agglomerates of highly disperse silver particles, the minimum dye detection concentration was at 10^−6^ mol/L. It was found that under hydrothermal conditions Ag films with bimodal particle size distribution were formed. When the synthesis temperature was increased to 120 and 140 °C, both an increase in the size of silver particles and the size of the gap between them were observed. The more intense SERS effect in the detection of methylene blue at low concentrations for Ag films grown in hydrothermal conditions at 100 °C was due to a more uniform microstructure, smaller particle and CSR sizes, as well as lower content of residual organic components. Thus, the obtained Ag thin-film nanostructures can be effectively used as SERS substrates for low concentrations detection of methylene blue and other analytes.

## Data Availability

Not applicable.
